# O4-2 Evaluate the health-enhancing physical activity policy of Rio de Janeiro: ‘Programa Academia Carioca'

**DOI:** 10.1093/eurpub/ckac094.026

**Published:** 2022-08-29

**Authors:** Junia Cardoso, Antoine Noel-Racine, José Augusto Guimarães de Oliveira, Paulo Mota, Jean-Marie Garbarino, Bernard Massiera, Anne Vuillemin, Rosangela Caetano, Claudia Coeli

**Affiliations:** 1 IESC, Universidade Federal do Rio de Janeiro, Rio de Janeiro, Brazil; 2 Université Côte d'Azur, LAMHESS, France; 3 CTPS, Secretaria Municipal de Saúde do Rio de Janeiro, Rio de Janeiro, Brazil; 4 IMS, Universidade do Estado do Rio de Janeiro, Rio de Janeiro, Brazil

**Keywords:** physical activity, health promotion, municipality, chronic diseases, primary health care

## Abstract

**Issue and problem:**

In Brazil, 47% of people are insufficiently physically active (Guthold et al, 2018). According to the Global Physical Activity Observatory, 13.2% of deaths in Brazil are caused by inactivity. Besides, Ding et al (2016) estimated the direct health cost attributable to physical inactivity in this country at 1.6 billion US dollars. Rio de Janeiro (RJ) is one of the largest cities in Brazil, with a high mortality rate (54.1%) from non-communicable diseases (NCDs) and substantial health inequalities (SIM/MRJ, 2019). As a large city, it reproduces a worldwide trend, which associates economic growth and urbanization with an unhealthy lifestyle and epidemic levels of obesity and remarkable physical inactivity (WHO, 2014).

**Problem description:**

The multifactorial context above described enables NCDs and challenges health protection systems. For this reason, the Municipal Health Department of RJ implemented since 2009 a Health-Enhancing Physical Activity (HEPA) policy: ‘Programa Academia Carioca' (PAC). This policy supports regular physical activity free of charge, in Primary Care Health Units (UAP). The Program combines the practice of physical activity associated with various educational and community activities. It includes Physical Education professionals who work in a multidisciplinary way. Therefore, this study aims to evaluate the implementation of this policy.

**Results (effects/changes):**

After 11 years, the PAC has 142,969 participants, 80% of whom have NCDs. Among its participants, blood pressure control was demonstrated in 90% of hypertensive patients; weight loss, and a 60% reduction in cardiovascular risk classification, and suspension of medication in 20% of users. Being part of the PAC resulted in greater use and understanding of the general services of UAP in 86% and 98% of participants, respectively. Detailed results are not available at that time but will be presented at the conference.

**Lessons:**

The PAC showed to be a successful tool to enhance health through physical activity at UAP. The use of trained and specialized professionals plays a fundamental key to develop educational and community actions in vulnerable groups.

**Main messages:**

The implementation of an ambitious and integrated HEPA policy, considering environmental and health factors, can produce more effective institutional responses to change this epidemiological scenario.

